# Crystal structure of 2,3-dimeth­oxy-5,6,7,8,13,13a-hexa­hydro-6a,8-di­aza­indeno­[2,1-*b*]phenanthrene methanol monosolvate

**DOI:** 10.1107/S2056989015013286

**Published:** 2015-07-15

**Authors:** Shahobiddin M. Adizov, Abdusalom Sh. Saidov, Kambarali K. Turgunov, Rasul Ya. Okmanov, Bakhodir Tashkhodjaev

**Affiliations:** a S.Yunusov Institute of the Chemistry of Plant Substances, Academy of Sciences of Uzbekistan, Mirzo Ulugbek Str. 77, Tashkent 100170, Uzbekistan

**Keywords:** crystal structure, indole alkaloid, iso­quinoline alkaloid, hydrogen bonding

## Abstract

The asymmetric unit of the title solvate, C_21_H_22_N_2_O_2_·CH_3_OH, contains one methanol solvent mol­ecule and one mol­ecule of the heterocycle that is built up by the fusion of four six-membered rings *A*, *C*, *D*, *E* and one five-membered ring *B*. The indole moiety (rings *A* and *B*) is essentially planar, with an r.m.s. deviation of 0.013 Å, whereas rings *C* and *D* adopt a twisted conformation with a *trans*-ring junction. In the crystal, two heterocyclic mol­ecules are associated with two methanol mol­ecules through mutual N—H⋯O and O—H⋯N hydrogen bonds, forming a centrosymmetric dimer.

## Related literature   

Synthetic details regarding the title compound were described by Saidov *et al.* (2014 ▸). For synthetic procedures of related compounds and their structures, see: Saidov *et al.* (2013 ▸). For another related crystal structure, see: Yu *et al.* (1995 ▸).
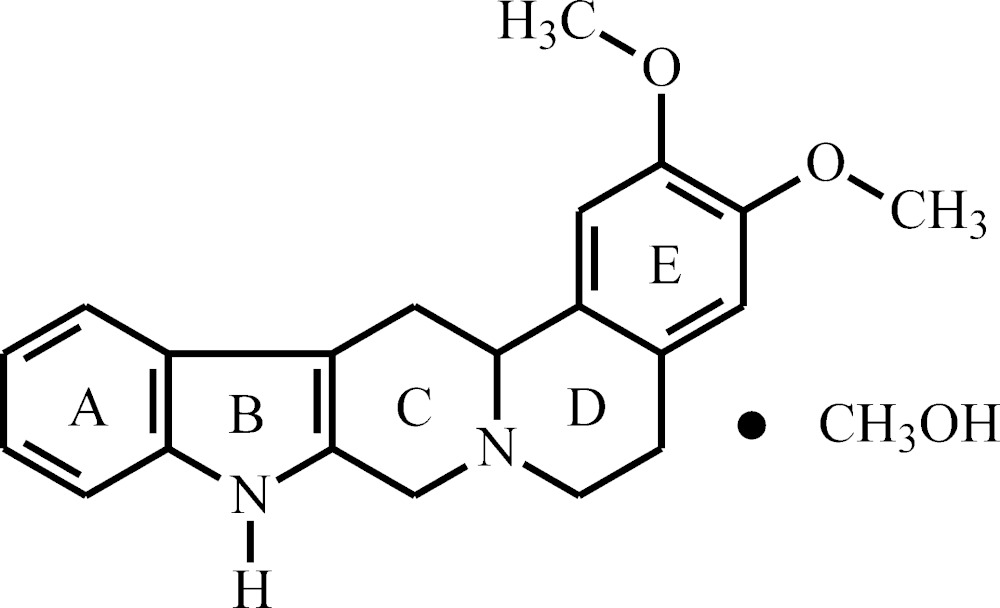


## Experimental   

### Crystal data  

C_21_H_22_N_2_O_2_·CH_4_O
*M* *_r_* = 366.45
Triclinic, 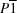

*a* = 7.6080 (3) Å
*b* = 11.8061 (5) Å
*c* = 12.3327 (5) Å
α = 65.242 (4)°
β = 73.956 (4)°
γ = 75.724 (4)°
*V* = 955.81 (7) Å^3^
*Z* = 2
Cu *K*α radiation
μ = 0.68 mm^−1^
*T* = 293 K
0.42 × 0.25 × 0.12 mm

### Data collection  

Oxford Diffraction Xcalibur Ruby diffractometer
Absorption correction: multi-scan (*CrysAlis PRO*; Oxford Diffraction, 2009 ▸) *T*_min_ = 0.843, *T*_max_ = 1.000
19864 measured reflections
3936 independent reflections
2889 reflections with *I* > 2σ(*I*)
*R*_int_ = 0.046

### Refinement  

*R*[*F* ^2^ > 2σ(*F* ^2^)] = 0.040
*wR*(*F* ^2^) = 0.120
*S* = 1.04
3936 reflections
256 parameters
H atoms treated by a mixture of independent and constrained refinement
Δρ_max_ = 0.17 e Å^−3^
Δρ_min_ = −0.17 e Å^−3^

### 

Data collection: *CrysAlis PRO* (Oxford Diffraction, 2009 ▸); cell refinement: *CrysAlis PRO*; data reduction: *CrysAlis PRO*; program(s) used to solve structure: *SHELXS97* (Sheldrick, 2008 ▸); program(s) used to refine structure: *SHELXL97* (Sheldrick, 2008 ▸); molecular graphics: *XP* in *SHELXTL* (Sheldrick, 2008 ▸); software used to prepare material for publication: *publCIF* (Westrip, 2010 ▸).

## Supplementary Material

Crystal structure: contains datablock(s) I, New_Global_Publ_Block. DOI: 10.1107/S2056989015013286/wm5184sup1.cif

Structure factors: contains datablock(s) I. DOI: 10.1107/S2056989015013286/wm5184Isup2.hkl

Supporting information file. DOI: 10.1107/S2056989015013286/wm5184Isup3.cml

**Fig. 1.**
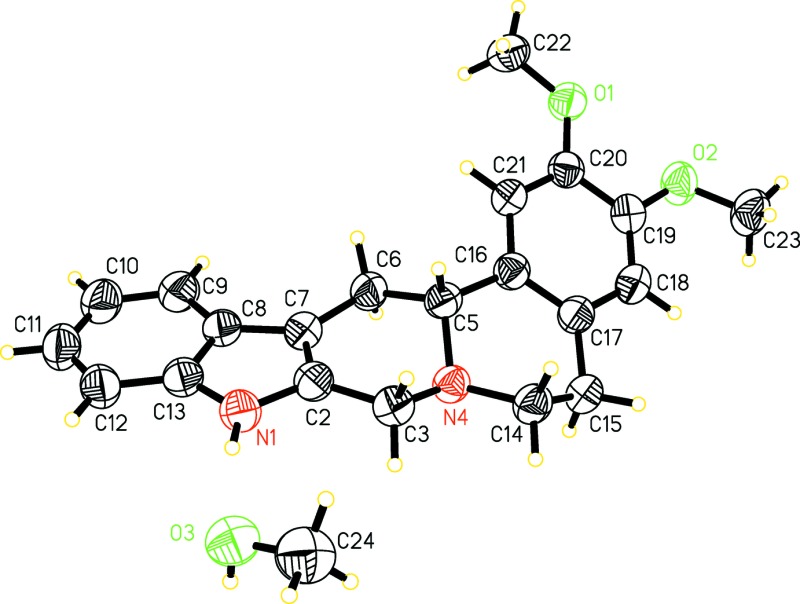
The molecular components of the title compound, with displacement ellipsoids drawn at the 50% probability level.

. DOI: 10.1107/S2056989015013286/wm5184fig1.tif
The mol­ecular components of the title compound, with displacement ellipsoids drawn at the 50% probability level.

**Fig. 2.**
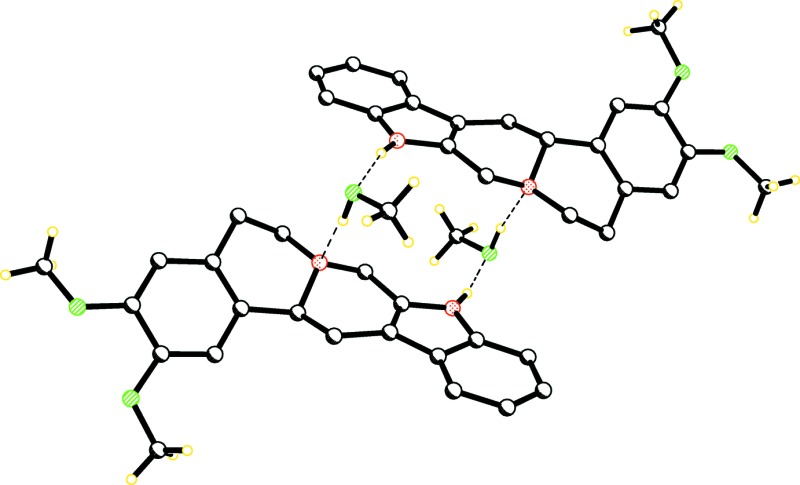
Hydrogen bonding between the molecular components, leading to the formation of dimers.

. DOI: 10.1107/S2056989015013286/wm5184fig2.tif
Hydrogen bonding between the mol­ecular components, leading to the formation of dimers.

CCDC reference: 1412105

Additional supporting information:  crystallographic information; 3D view; checkCIF report

## Figures and Tables

**Table 1:** Hydrogen-bond geometry (, )

*D*H*A*	*D*H	H*A*	*D* *A*	*D*H*A*
N1H1*A*O3	0.907(19)	1.982(19)	2.880(2)	170.1(16)
O3H3N4^i^	0.91(2)	1.95(2)	2.840(2)	165(2)

Symmetry code: (i) 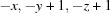.

## supplementary crystallographic information

### S1. Synthesis and crystallization

The synthesis of the title compound was described previously (Saidov *et al.*, 2014). Orange crystals were obtained in a methanol:chloro­form 1:1 (*v*/*v*) mixture by slow evaporation of the solvent at room temperature.

### S2. Refinement

Carbon-bound H atoms were placed geometrically and treated as riding on their parent atoms, with C—H = 0.93 Å (aromatic), 0.97 Å (methylen) or 0.96 Å (methyl) and were refined with *U*_iso_(H) = 1.2*U*_eq_(C) for aromatic and methyl­ene hydrogens and *U*_iso_(H) = 1.5*U*_eq_(C) for methyl H atoms. The N- and O-bound H atoms were located in a difference Fourier map and were refined freely.

### Crystal data

**Table:**

C_21_H_22_N_2_O_2_·CH_4_O	*Z* = 2
*M**_r_* = 366.45	*F*(000) = 392
Triclinic, *P*1	*D*_x_ = 1.273 Mg m^−^^3^
Hall symbol: -P 1	Melting point: 393(2) K
*a* = 7.6080 (3) Å	Cu *K*α radiation, λ = 1.54184 Å
*b* = 11.8061 (5) Å	Cell parameters from 7310 reflections
*c* = 12.3327 (5) Å	θ = 4.0–75.6°
α = 65.242 (4)°	µ = 0.68 mm^−^^1^
β = 73.956 (4)°	*T* = 293 K
γ = 75.724 (4)°	Prism, orange
*V* = 955.81 (7) Å^3^	0.42 × 0.25 × 0.12 mm

### Data collection

**Table:**

Oxford Diffraction Xcalibur Ruby diffractometer	3936 independent reflections
Radiation source: Enhance (Cu) X-ray Source	2889 reflections with *I* > 2σ(*I*)
Graphite monochromator	*R*_int_ = 0.046
Detector resolution: 10.2576 pixels mm^-1^	θ_max_ = 75.8°, θ_min_ = 4.0°
ω scans	*h* = −9→8
Absorption correction: multi-scan (*CrysAlis PRO*; Oxford Diffraction, 2009)	*k* = −14→14
*T*_min_ = 0.843, *T*_max_ = 1.000	*l* = −15→15
19864 measured reflections	

### Refinement

**Table:**

Refinement on *F*^2^	Secondary atom site location: difference Fourier map
Least-squares matrix: full	Hydrogen site location: inferred from neighbouring sites
*R*[*F*^2^ > 2σ(*F*^2^)] = 0.040	H atoms treated by a mixture of independent and constrained refinement
*wR*(*F*^2^) = 0.120	*w* = 1/[σ^2^(*F*_o_^2^) + (0.069*P*)^2^ + 0.0547*P*] where *P* = (*F*_o_^2^ + 2*F*_c_^2^)/3
*S* = 1.04	(Δ/σ)_max_ = 0.002
3936 reflections	Δρ_max_ = 0.17 e Å^−^^3^
256 parameters	Δρ_min_ = −0.17 e Å^−^^3^
0 restraints	Extinction correction: *SHELXL97* (Sheldrick, 2008), Fc^*^=kFc[1+0.001xFc^2^λ^3^/sin(2θ)]^-1/4^
Primary atom site location: structure-invariant direct methods	Extinction coefficient: 0.0025 (6)

### Special details

**Table:**

Geometry. All e.s.d.'s (except the e.s.d. in the dihedral angle between two l.s. planes) are estimated using the full covariance matrix. The cell e.s.d.'s are taken into account individually in the estimation of e.s.d.'s in distances, angles and torsion angles; correlations between e.s.d.'s in cell parameters are only used when they are defined by crystal symmetry. An approximate (isotropic) treatment of cell e.s.d.'s is used for estimating e.s.d.'s involving l.s. planes.
Refinement. Refinement of *F*^2^ against ALL reflections. The weighted *R*-factor *wR* and goodness of fit *S* are based on *F*^2^, conventional *R*-factors *R* are based on *F*, with *F* set to zero for negative *F*^2^. The threshold expression of *F*^2^ > σ(*F*^2^) is used only for calculating *R*-factors(gt) *etc*. and is not relevant to the choice of reflections for refinement. *R*-factors based on *F*^2^ are statistically about twice as large as those based on *F*, and *R*- factors based on ALL data will be even larger.

### Fractional atomic coordinates and isotropic or equivalent isotropic displacement parameters (Å^2^)

**Table:**

	*x*	*y*	*z*	*U*_iso_*/*U*_eq_	
O1	0.58933 (14)	0.05796 (10)	1.11979 (9)	0.0544 (3)	
O2	0.27998 (15)	0.08093 (10)	1.27098 (9)	0.0582 (3)	
O3	0.10908 (17)	0.72865 (11)	0.27339 (11)	0.0653 (3)	
N1	0.30388 (18)	0.48439 (12)	0.39691 (11)	0.0499 (3)	
N4	0.13746 (15)	0.41753 (10)	0.72574 (10)	0.0430 (3)	
C2	0.2862 (2)	0.43976 (13)	0.52151 (13)	0.0457 (3)	
C3	0.1738 (2)	0.50958 (13)	0.59986 (12)	0.0462 (3)	
H3A	0.2404	0.5723	0.5951	0.055*	
H3B	0.0581	0.5523	0.5730	0.055*	
C5	0.31440 (18)	0.34322 (13)	0.76212 (12)	0.0437 (3)	
H5A	0.4035	0.4020	0.7338	0.052*	
C6	0.3879 (2)	0.25152 (14)	0.69509 (13)	0.0497 (3)	
H6A	0.5147	0.2147	0.7036	0.060*	
H6B	0.3139	0.1838	0.7305	0.060*	
C7	0.37932 (19)	0.32112 (14)	0.56320 (13)	0.0464 (3)	
C8	0.45916 (19)	0.28619 (14)	0.45984 (13)	0.0475 (3)	
C9	0.5643 (2)	0.17724 (16)	0.44332 (16)	0.0590 (4)	
H9A	0.5961	0.1063	0.5097	0.071*	
C10	0.6200 (2)	0.17645 (19)	0.32772 (17)	0.0669 (5)	
H10A	0.6895	0.1042	0.3163	0.080*	
C11	0.5741 (2)	0.28218 (19)	0.22718 (16)	0.0654 (5)	
H11A	0.6147	0.2792	0.1499	0.078*	
C12	0.4700 (2)	0.39083 (17)	0.23980 (14)	0.0578 (4)	
H12A	0.4405	0.4612	0.1724	0.069*	
C13	0.4104 (2)	0.39180 (15)	0.35692 (13)	0.0493 (3)	
C14	0.0333 (2)	0.48294 (13)	0.80799 (13)	0.0485 (3)	
H14A	−0.0728	0.5392	0.7760	0.058*	
H14B	0.1113	0.5329	0.8141	0.058*	
C15	−0.0305 (2)	0.38641 (14)	0.93244 (13)	0.0502 (4)	
H15A	−0.0932	0.4286	0.9884	0.060*	
H15B	−0.1174	0.3418	0.9273	0.060*	
C16	0.29541 (19)	0.27694 (13)	0.89861 (12)	0.0438 (3)	
C17	0.13271 (19)	0.29376 (13)	0.97944 (13)	0.0455 (3)	
C18	0.1258 (2)	0.22773 (14)	1.10490 (13)	0.0493 (3)	
H18A	0.0162	0.2376	1.1590	0.059*	
C19	0.2769 (2)	0.14886 (13)	1.14984 (13)	0.0473 (3)	
C20	0.44399 (19)	0.13467 (13)	1.06780 (13)	0.0452 (3)	
C21	0.45059 (19)	0.19815 (13)	0.94490 (13)	0.0456 (3)	
H21A	0.5607	0.1887	0.8909	0.055*	
C22	0.7633 (2)	0.04838 (16)	1.04086 (15)	0.0574 (4)	
H22A	0.8558	−0.0020	1.0883	0.086*	
H22B	0.7961	0.1311	0.9928	0.086*	
H22C	0.7555	0.0096	0.9881	0.086*	
C23	0.1439 (3)	0.12453 (19)	1.35495 (15)	0.0724 (5)	
H23A	0.1714	0.0793	1.4350	0.109*	
H23B	0.0246	0.1109	1.3549	0.109*	
H23C	0.1433	0.2130	1.3319	0.109*	
C24	0.0459 (3)	0.81552 (19)	0.3320 (2)	0.0782 (5)	
H24A	−0.0599	0.7900	0.3951	0.117*	
H24B	0.0122	0.8979	0.2737	0.117*	
H24C	0.1425	0.8178	0.3670	0.117*	
H1A	0.254 (2)	0.5621 (17)	0.3504 (15)	0.060 (5)*	
H3	0.014 (3)	0.694 (2)	0.2747 (18)	0.085 (6)*	

### Atomic displacement parameters (Å^2^)

**Table:**

	*U*^11^	*U*^22^	*U*^33^	*U*^12^	*U*^13^	*U*^23^
O1	0.0513 (6)	0.0577 (6)	0.0479 (6)	0.0017 (5)	−0.0120 (4)	−0.0179 (5)
O2	0.0644 (7)	0.0589 (6)	0.0423 (6)	−0.0024 (5)	−0.0066 (5)	−0.0166 (5)
O3	0.0602 (7)	0.0617 (7)	0.0753 (8)	−0.0148 (6)	−0.0117 (6)	−0.0251 (6)
N1	0.0513 (7)	0.0490 (7)	0.0448 (7)	−0.0092 (6)	−0.0086 (5)	−0.0129 (6)
N4	0.0425 (6)	0.0410 (6)	0.0441 (6)	−0.0033 (5)	−0.0078 (5)	−0.0168 (5)
C2	0.0443 (8)	0.0471 (7)	0.0443 (7)	−0.0111 (6)	−0.0063 (6)	−0.0151 (6)
C3	0.0470 (8)	0.0412 (7)	0.0476 (8)	−0.0066 (6)	−0.0094 (6)	−0.0141 (6)
C5	0.0382 (7)	0.0464 (7)	0.0456 (7)	−0.0057 (6)	−0.0070 (6)	−0.0176 (6)
C6	0.0483 (8)	0.0512 (8)	0.0455 (8)	0.0031 (6)	−0.0102 (6)	−0.0195 (6)
C7	0.0430 (8)	0.0499 (8)	0.0452 (7)	−0.0058 (6)	−0.0068 (6)	−0.0186 (6)
C8	0.0409 (8)	0.0554 (8)	0.0490 (8)	−0.0098 (6)	−0.0074 (6)	−0.0217 (7)
C9	0.0531 (9)	0.0632 (10)	0.0638 (10)	0.0000 (7)	−0.0114 (7)	−0.0318 (8)
C10	0.0594 (10)	0.0801 (12)	0.0719 (12)	−0.0031 (9)	−0.0079 (8)	−0.0462 (10)
C11	0.0586 (10)	0.0928 (13)	0.0580 (10)	−0.0202 (9)	−0.0002 (8)	−0.0432 (10)
C12	0.0548 (9)	0.0743 (11)	0.0481 (8)	−0.0198 (8)	−0.0061 (7)	−0.0238 (8)
C13	0.0419 (8)	0.0588 (9)	0.0501 (8)	−0.0149 (6)	−0.0055 (6)	−0.0215 (7)
C14	0.0467 (8)	0.0461 (8)	0.0540 (8)	−0.0009 (6)	−0.0090 (6)	−0.0240 (7)
C15	0.0432 (8)	0.0561 (8)	0.0513 (8)	−0.0028 (6)	−0.0048 (6)	−0.0256 (7)
C16	0.0422 (7)	0.0452 (7)	0.0456 (7)	−0.0079 (6)	−0.0063 (6)	−0.0194 (6)
C17	0.0432 (8)	0.0470 (7)	0.0478 (8)	−0.0071 (6)	−0.0067 (6)	−0.0206 (6)
C18	0.0466 (8)	0.0526 (8)	0.0470 (8)	−0.0082 (6)	−0.0010 (6)	−0.0219 (7)
C19	0.0534 (9)	0.0460 (7)	0.0428 (7)	−0.0087 (6)	−0.0073 (6)	−0.0178 (6)
C20	0.0466 (8)	0.0438 (7)	0.0481 (8)	−0.0045 (6)	−0.0115 (6)	−0.0201 (6)
C21	0.0408 (7)	0.0507 (8)	0.0457 (8)	−0.0056 (6)	−0.0054 (6)	−0.0212 (6)
C22	0.0457 (8)	0.0602 (9)	0.0602 (9)	−0.0036 (7)	−0.0108 (7)	−0.0192 (8)
C23	0.0702 (12)	0.0829 (12)	0.0440 (9)	0.0036 (9)	−0.0024 (8)	−0.0185 (9)
C24	0.0810 (13)	0.0703 (12)	0.0934 (14)	−0.0119 (10)	−0.0234 (11)	−0.0362 (11)

### Geometric parameters (Å, º)

**Table:**

O1—C20	1.3658 (17)	C10—H10A	0.9300
O1—C22	1.4207 (18)	C11—C12	1.376 (2)
O2—C19	1.3720 (17)	C11—H11A	0.9300
O2—C23	1.4143 (19)	C12—C13	1.393 (2)
O3—C24	1.411 (2)	C12—H12A	0.9300
O3—H3	0.91 (2)	C14—C15	1.512 (2)
N1—C13	1.3743 (19)	C14—H14A	0.9700
N1—C2	1.3782 (18)	C14—H14B	0.9700
N1—H1A	0.907 (18)	C15—C17	1.507 (2)
N4—C14	1.4706 (17)	C15—H15A	0.9700
N4—C3	1.4716 (17)	C15—H15B	0.9700
N4—C5	1.4849 (17)	C16—C17	1.3885 (19)
C2—C7	1.355 (2)	C16—C21	1.4040 (19)
C2—C3	1.4856 (19)	C17—C18	1.403 (2)
C3—H3A	0.9700	C18—C19	1.373 (2)
C3—H3B	0.9700	C18—H18A	0.9300
C5—C16	1.5115 (19)	C19—C20	1.412 (2)
C5—C6	1.5374 (19)	C20—C21	1.3735 (19)
C5—H5A	0.9800	C21—H21A	0.9300
C6—C7	1.4959 (19)	C22—H22A	0.9600
C6—H6A	0.9700	C22—H22B	0.9600
C6—H6B	0.9700	C22—H22C	0.9600
C7—C8	1.433 (2)	C23—H23A	0.9600
C8—C9	1.399 (2)	C23—H23B	0.9600
C8—C13	1.418 (2)	C23—H23C	0.9600
C9—C10	1.374 (2)	C24—H24A	0.9600
C9—H9A	0.9300	C24—H24B	0.9600
C10—C11	1.395 (3)	C24—H24C	0.9600
			
C20—O1—C22	117.34 (11)	C12—C13—C8	121.50 (15)
C19—O2—C23	116.67 (12)	N4—C14—C15	109.21 (11)
C24—O3—H3	111.0 (13)	N4—C14—H14A	109.8
C13—N1—C2	108.22 (13)	C15—C14—H14A	109.8
C13—N1—H1A	126.7 (11)	N4—C14—H14B	109.8
C2—N1—H1A	125.0 (11)	C15—C14—H14B	109.8
C14—N4—C3	109.92 (10)	H14A—C14—H14B	108.3
C14—N4—C5	111.77 (11)	C17—C15—C14	110.14 (12)
C3—N4—C5	109.95 (10)	C17—C15—H15A	109.6
C7—C2—N1	110.50 (13)	C14—C15—H15A	109.6
C7—C2—C3	124.68 (13)	C17—C15—H15B	109.6
N1—C2—C3	124.76 (13)	C14—C15—H15B	109.6
N4—C3—C2	107.82 (11)	H15A—C15—H15B	108.1
N4—C3—H3A	110.1	C17—C16—C21	119.03 (13)
C2—C3—H3A	110.1	C17—C16—C5	122.61 (13)
N4—C3—H3B	110.1	C21—C16—C5	118.33 (12)
C2—C3—H3B	110.1	C16—C17—C18	119.08 (13)
H3A—C3—H3B	108.5	C16—C17—C15	120.00 (13)
N4—C5—C16	112.57 (11)	C18—C17—C15	120.86 (12)
N4—C5—C6	108.21 (11)	C19—C18—C17	121.73 (13)
C16—C5—C6	112.62 (11)	C19—C18—H18A	119.1
N4—C5—H5A	107.7	C17—C18—H18A	119.1
C16—C5—H5A	107.7	O2—C19—C18	124.92 (13)
C6—C5—H5A	107.7	O2—C19—C20	115.89 (13)
C7—C6—C5	109.45 (12)	C18—C19—C20	119.19 (13)
C7—C6—H6A	109.8	O1—C20—C21	125.15 (13)
C5—C6—H6A	109.8	O1—C20—C19	115.63 (12)
C7—C6—H6B	109.8	C21—C20—C19	119.21 (13)
C5—C6—H6B	109.8	C20—C21—C16	121.71 (13)
H6A—C6—H6B	108.2	C20—C21—H21A	119.1
C2—C7—C8	106.95 (13)	C16—C21—H21A	119.1
C2—C7—C6	121.66 (13)	O1—C22—H22A	109.5
C8—C7—C6	131.38 (13)	O1—C22—H22B	109.5
C9—C8—C13	118.97 (14)	H22A—C22—H22B	109.5
C9—C8—C7	134.61 (15)	O1—C22—H22C	109.5
C13—C8—C7	106.42 (13)	H22A—C22—H22C	109.5
C10—C9—C8	119.10 (17)	H22B—C22—H22C	109.5
C10—C9—H9A	120.5	O2—C23—H23A	109.5
C8—C9—H9A	120.5	O2—C23—H23B	109.5
C9—C10—C11	121.15 (17)	H23A—C23—H23B	109.5
C9—C10—H10A	119.4	O2—C23—H23C	109.5
C11—C10—H10A	119.4	H23A—C23—H23C	109.5
C12—C11—C10	121.47 (16)	H23B—C23—H23C	109.5
C12—C11—H11A	119.3	O3—C24—H24A	109.5
C10—C11—H11A	119.3	O3—C24—H24B	109.5
C11—C12—C13	117.78 (16)	H24A—C24—H24B	109.5
C11—C12—H12A	121.1	O3—C24—H24C	109.5
C13—C12—H12A	121.1	H24A—C24—H24C	109.5
N1—C13—C12	130.61 (15)	H24B—C24—H24C	109.5
N1—C13—C8	107.89 (13)		
			
C13—N1—C2—C7	0.40 (16)	C7—C8—C13—N1	−1.05 (16)
C13—N1—C2—C3	−177.03 (13)	C9—C8—C13—C12	−2.2 (2)
C14—N4—C3—C2	177.49 (12)	C7—C8—C13—C12	178.47 (13)
C5—N4—C3—C2	54.04 (14)	C3—N4—C14—C15	170.60 (11)
C7—C2—C3—N4	−16.43 (19)	C5—N4—C14—C15	−67.03 (15)
N1—C2—C3—N4	160.63 (13)	N4—C14—C15—C17	56.04 (16)
C14—N4—C5—C16	39.91 (15)	N4—C5—C16—C17	−5.02 (19)
C3—N4—C5—C16	162.27 (11)	C6—C5—C16—C17	−127.70 (15)
C14—N4—C5—C6	165.02 (11)	N4—C5—C16—C21	176.81 (12)
C3—N4—C5—C6	−72.62 (13)	C6—C5—C16—C21	54.14 (17)
N4—C5—C6—C7	47.51 (15)	C21—C16—C17—C18	−2.5 (2)
C16—C5—C6—C7	172.59 (11)	C5—C16—C17—C18	179.38 (13)
N1—C2—C7—C8	−1.06 (17)	C21—C16—C17—C15	174.74 (13)
C3—C2—C7—C8	176.37 (13)	C5—C16—C17—C15	−3.4 (2)
N1—C2—C7—C6	178.11 (13)	C14—C15—C17—C16	−21.95 (19)
C3—C2—C7—C6	−4.5 (2)	C14—C15—C17—C18	155.21 (13)
C5—C6—C7—C2	−11.52 (19)	C16—C17—C18—C19	1.3 (2)
C5—C6—C7—C8	167.41 (14)	C15—C17—C18—C19	−175.89 (14)
C2—C7—C8—C9	−177.88 (16)	C23—O2—C19—C18	−21.5 (2)
C6—C7—C8—C9	3.1 (3)	C23—O2—C19—C20	158.32 (15)
C2—C7—C8—C13	1.28 (16)	C17—C18—C19—O2	−179.63 (13)
C6—C7—C8—C13	−177.76 (14)	C17—C18—C19—C20	0.6 (2)
C13—C8—C9—C10	1.1 (2)	C22—O1—C20—C21	3.5 (2)
C7—C8—C9—C10	−179.79 (16)	C22—O1—C20—C19	−175.59 (13)
C8—C9—C10—C11	0.2 (3)	O2—C19—C20—O1	−1.91 (19)
C9—C10—C11—C12	−0.6 (3)	C18—C19—C20—O1	177.89 (12)
C10—C11—C12—C13	−0.4 (2)	O2—C19—C20—C21	178.95 (12)
C2—N1—C13—C12	−179.03 (15)	C18—C19—C20—C21	−1.3 (2)
C2—N1—C13—C8	0.44 (16)	O1—C20—C21—C16	−179.02 (13)
C11—C12—C13—N1	−178.76 (15)	C19—C20—C21—C16	0.0 (2)
C11—C12—C13—C8	1.8 (2)	C17—C16—C21—C20	1.8 (2)
C9—C8—C13—N1	178.27 (13)	C5—C16—C21—C20	−179.92 (13)

### Hydrogen-bond geometry (Å, º)

**Table:**

*D*—H···*A*	*D*—H	H···*A*	*D*···*A*	*D*—H···*A*
N1—H1*A*···O3	0.907 (19)	1.982 (19)	2.880 (2)	170.1 (16)
O3—H3···N4^i^	0.91 (2)	1.95 (2)	2.840 (2)	165 (2)

Symmetry code: (i) −*x*, −*y*+1, −*z*+1.
