# Supplementary figures and images for: Crystal structure of 2,3-dimeth­oxy-5,6,7,8,13,13a-hexa­hydro-6a,8-di­aza­indeno­[2,1-b]phenanthrene methanol monosolvate
Source: Acta Crystallogr E Crystallogr Commun. 2015 Jul 15;71(Pt 8):o574–5. doi: 10.1107/S2056989015013286 (PMC4571404; doi:10.1107/S2056989015013286)

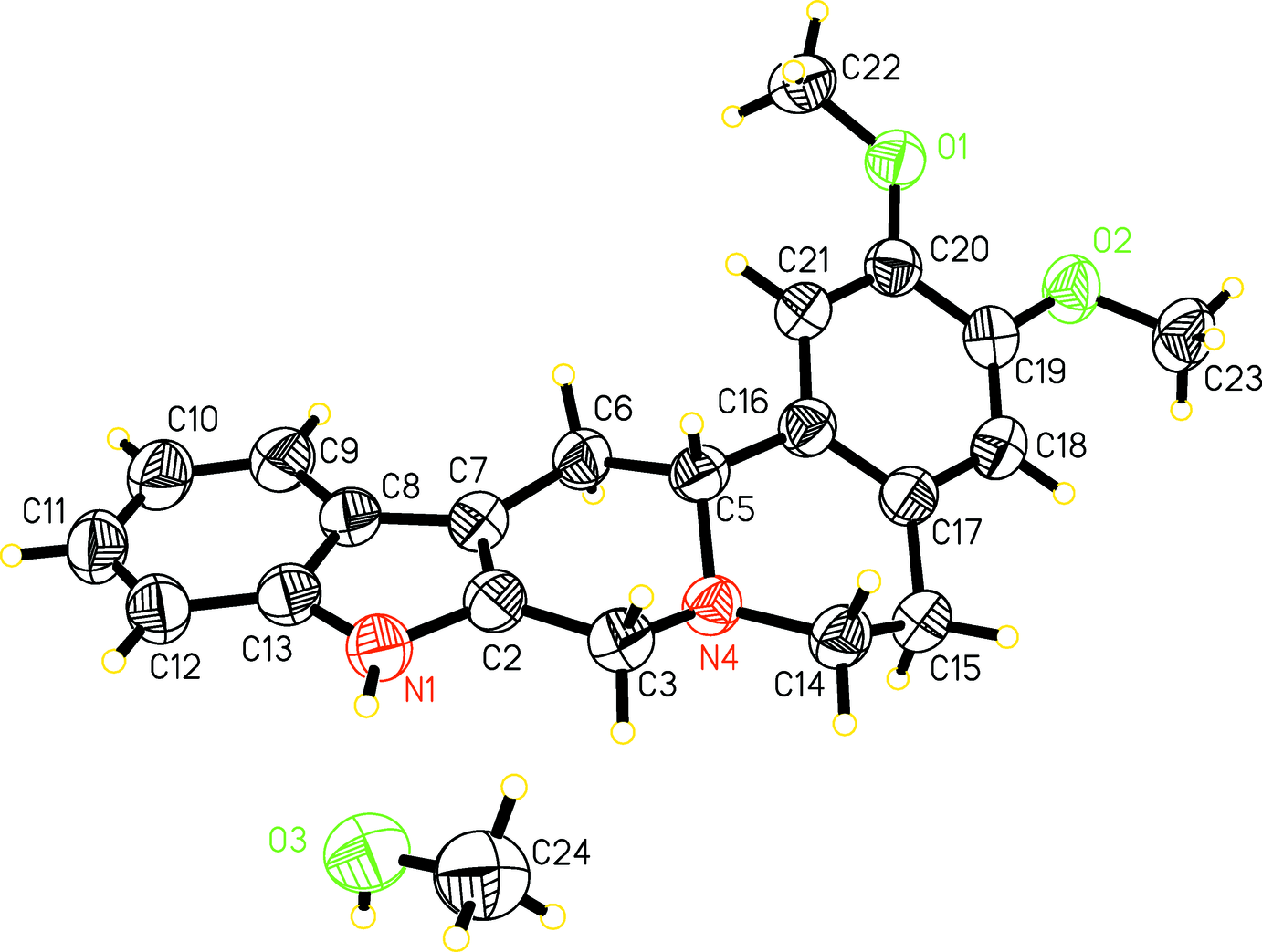

Supplement: Supplementary file 4 [file e-71-0o574-fig1.tif]

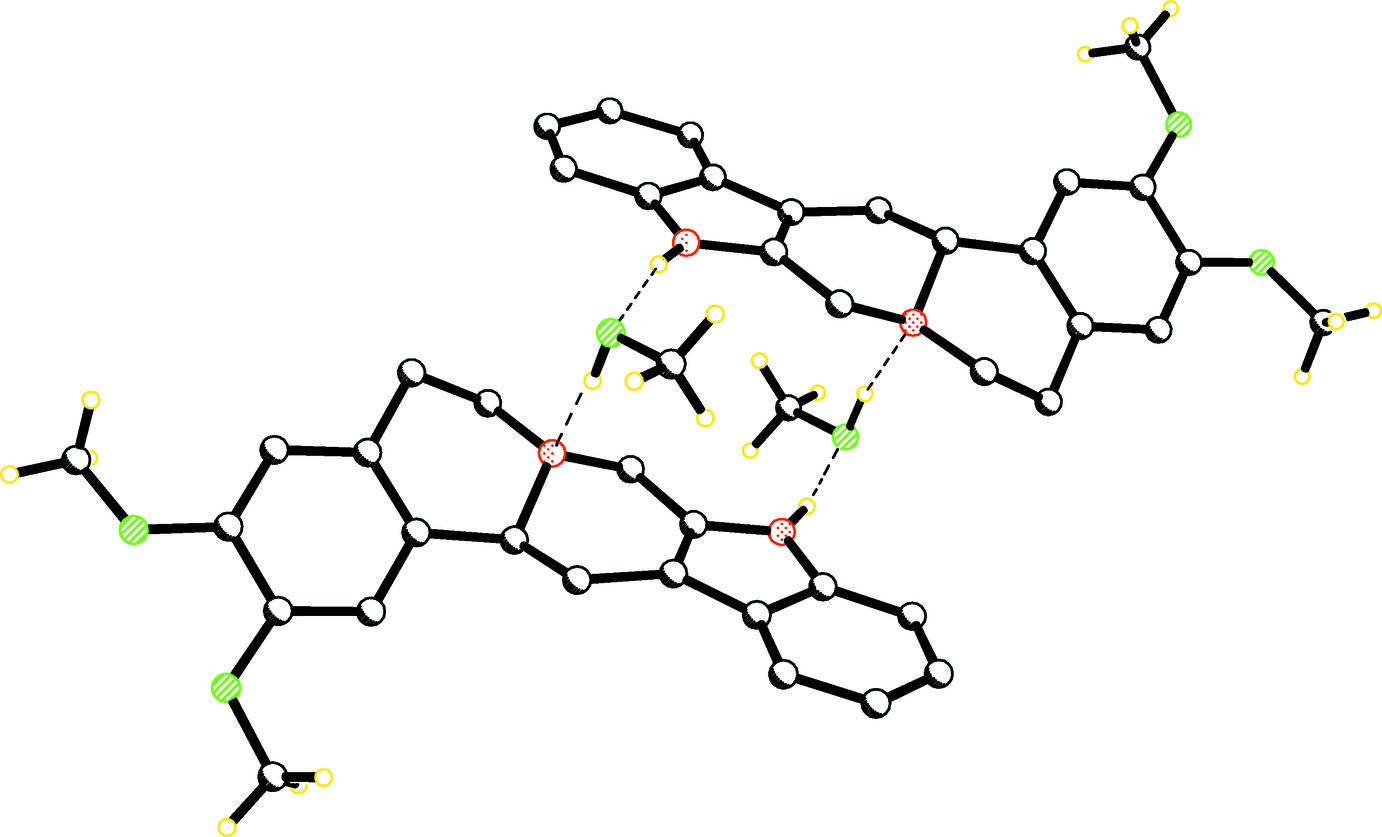

Supplement: Supplementary file 5 [file e-71-0o574-fig2.tif]
